# Silicon carbide X-ray beam position monitors for synchrotron applications

**DOI:** 10.1107/S1600577518014248

**Published:** 2019-01-01

**Authors:** Selamnesh Nida, Alexander Tsibizov, Thomas Ziemann, Judith Woerle, Andy Moesch, Clemens Schulze-Briese, Claude Pradervand, Salvatore Tudisco, Hans Sigg, Oliver Bunk, Ulrike Grossner, Massimo Camarda

**Affiliations:** a Advanced Power Semiconductor Laboratory, ETH Zurich, Zurich, Switzerland; b Paul Scherrer Institute, Villigen, Switzerland; c DECTRIS Ltd, Baden-Daettwil, Switzerland; d INFN – Laboratori Nazionali del Sud, Catania, Italy

**Keywords:** beam position monitors, silicon carbide, radiation detector, beamline instrumentation, X-rays

## Abstract

In this work, the performance of thin silicon carbide membranes as material for radiation hard X-ray beam position monitors (XBPMs) is investigated. Thermal and electrical behavior of XBPMs made from thin silicon carbide membranes and single-crystal diamond is compared using finite-element simulations. Fabricated silicon carbide devices are also compared with a 12 µm commercial polycrystalline diamond XBPM at the Swiss Light Source at the Paul Scherrer Institute. Results show that silicon carbide devices can reach equivalent transparencies while showing improved linearity, dynamics and signal-to-noise ratio compared with commercial polycrystalline diamond XBPMs. Given the obtained results and availability of electronic-grade epitaxies on up to 6 inch wafers, it is expected that silicon carbide can substitute for diamond in most beam monitoring applications, whereas diamond, owing to its lower absorption, could remain the material of choice in cases of extreme X-ray power densities, such as pink and white beams.

## Abstract 2

The viability of thin 4H-SiC membrane X-ray beam position monitors in synchrotrons is investigated. Devices are fabricated and show improved linearity, dynamics and signal-to-noise ratio compared with commercial polycrystalline diamond X-ray beam position monitors.

## 1. Introduction   

Synchrotron light sources deliver X-ray beams with high brilliance to endstations, where experiments from macromolecular crystallography to scanning X-ray nanoprobe are conducted (Owen *et al.*, 2016 ▸). These applications benefit from highly transparent, compact, fast and reliable X-ray beam position monitors (XBPMs) with high lateral resolutions, capable of withstanding high power densities and high radiation doses. Such devices enable precise determination of intensity, position and, in some cases, shape of the beam. This can be used to establish a feedback loop with beamline optics improving beam position stability.

State-of-the-art XBPMs can be divided into three types: (i) peripheral, non-destructive in-line XBPMs such as blade monitors which intercept only the outer fringes; (ii) on-axis, destructive XBPMs such as fluorescent screens; and (iii) on-axis, non-destructive in-line XBPMs with high transparency to the beam (Schulze-Briese *et al.*, 2001 ▸; Leban *et al.*, 2010 ▸). Peripheral XBPMs allow minimal beam interference but suffer from low lateral resolution, high sensitivity to external noise, and systematic errors in the case of non-Gaussian beams. In-line, destructive XBPMs interfere with the beam and are operated only during beamline commissioning and set up (Bunk *et al.*, 2005 ▸). However, to perform automatic optics corrections during operation, continuous monitoring with high lateral resolution and good signal-to-noise ratio is needed. This increases the quality of beam delivered to the endstations and thus the quality of experimental data. To this end, non-destructive, in-line XBPMs are needed.

The main drawbacks of in-line XBPMs are residual interference with the beam and degradation due to heat load and radiation. To minimize these drawbacks, transparency, high-temperature stability and radiation hardness are primary requirements for this category of monitors. Linearity and fast dynamics are also important to maintain a stable feedback. This has driven research in wide-band-gap semiconductors for radiation monitor applications (Schulze-Briese *et al.*, 2001 ▸).

Diamond is the material of choice among wide-band-gap semiconductors due to its excellent transparency, radiation hardness and high thermal conductivity (Schulze-Briese *et al.*, 2001 ▸; Smedley *et al.*, 2011 ▸; Muller *et al.*, 2012 ▸; Marinelli *et al.*, 2012 ▸; Desjardins *et al.*, 2014 ▸; Zhou *et al.*, 2015 ▸; Williams *et al.*, 2016 ▸; Griesmayer *et al.*, 2016 ▸). Single-crystal diamond (sc-diamond) as well as polycrystalline diamond on silicon (pc-diamond, or CVD diamond) XBPMs are now commercially available (DECTRIS, CIVIDEC). The desired device properties of sc-diamond are not obtained on a commercial scale due to the use of thick substrates with high absorption, and availability of samples only smaller than 10 mm × 10 mm (Khmelnitskiy, 2015 ▸). Improving transparency by thinning down the thick substrates and fabricating membranes encounters challenges. Reactive ion etching is tested but results in too high (>50%) thickness non-uniformity (Desjardins *et al.*, 2014 ▸).

A thin pc-diamond membrane grown on silicon is highly transparent. However, polycrystalline material has defects and grain boundaries that result in slow dynamics and non-linear­ities (Bergonzo *et al.*, 2006 ▸). The difference in thermal expansion between a thin diamond film and a thick Si-substrate also gives rise to wafer bowing, thereby limiting the wafer size to 3 inch (RIGI, DECTRIS). A high density of defects also reduces reproducibility hindering industrialization of the pc-diamond devices.

XBPMs made of silicon carbide would provide high thermal conductivity and inertness as their diamond counterparts (Desjardins *et al.*, 2014 ▸). Furthermore, electronic-grade single-crystal 4H-SiC wafers with much lower defects densities than diamond are available up to a diameter of 6 inch, avoiding the bottlenecks of diamond technology. Thus, this technologically mature wide-bandgap material shows great promise for next-generation industrial XBPMs.

Nevertheless, due to difficulties in fabricating micrometre thin active layers, 4H-SiC has never been considered as a candidate for XBPMs. Recently, it was shown that electrochemical etching in HF-based solutions could selectively remove the highly doped 4H-SiC substrate with an etch stop on the low-doped epitaxial layers (Dahal *et al.*, 2017 ▸). This method is used in our study to fabricate XBPMs on thin epitaxial membranes on 4H-SiC substrates. Fabricated devices are then tested and compared with a commercial 12 µm pc-diamond XBPM at the Swiss Light Source (SLS) at the Paul Scherrer Institute (PSI). The theoretical thermal and electrical behaviors of diamond and 4H-SiC XBPMs are described in the next section followed by the experimental results on the fabricated devices.

## 2. Device simulations   

### 2.1. Thermal simulations   

Diamond exhibits a high transparency to X-ray beams, and has a large thermal conductivity as well as low thermal expansion coefficient. As a result, it withstands high-brilliance X-ray beams with minimal degradation. 4H-SiC, on the other hand, has about ten times the absorption (650 *versus* 70 µm attenuation length at 8 keV) and half of the thermal conductivity of diamond (3.7 *versus* 22 W cm^−1^ K^−1^) (Henke *et al.*, 1993 ▸; Yu *et al.*, 2001 ▸). Because of this, it is important to investigate the thermal response of 4H-SiC XBPMs under high-brilliance pink beams from synchrotron light sources and free-electron lasers (XFEL) to assess their reliability in the different applications.

**Figure 1:**
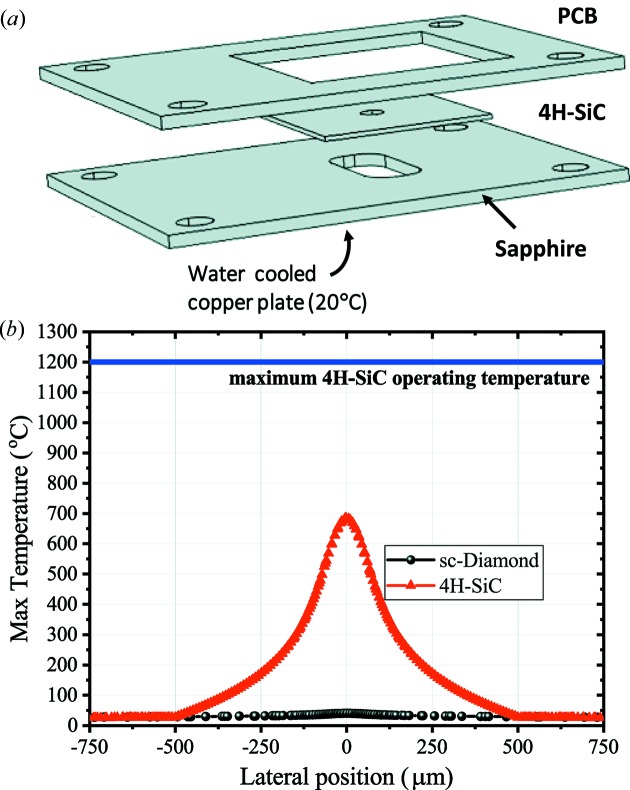
(*a*) Components of the full chip: the XBPM device, a sapphire plate, which is in contact with a water-cooled copper plate, and the printed circuit board (PCB). The PCB is placed on top for connections towards the XBPM and the readout system. (*b*) Simulated temperature profile across the 4H-SiC XBPM with the beam centered at the origin. As can be seen, already at 375 µm, the temperature drops below 100°C.

COMSOL 5.3 (COMSOL *Multiphysics*; https://www.comsol.com) is used in this report to calculate the thermo-mechanical response of a full chip, sensor plus packaging, under different high-brilliance beams. The fully packaged device considered in this study consists of a 1 mm sapphire plate, the XBPM and a 1.2 mm Rogers RO4003 printed circuit board (PCB) for readout [see Fig. 1(*a*) ▸]. The entire bottom of the sapphire plate is in contact with a water-cooled copper plate held at 20°C. A 5 mm × 12 mm opening is made in the sapphire plate for the beam. However, as can be seen in Fig. 1(*b*) ▸, the temperature already reaches 100°C within 500 µm, *i.e.* within the 4H-SiC substrate. All other surfaces are assumed to be fully insulated to represent a standard operation in vacuum.

To compare the temperature profiles of 4H-SiC and diamond under equivalent transparencies, a 10 µm- and 1.075 µm-thick membrane is assumed for diamond and 4H-SiC, respectively (Henke *et al.*, 1993 ▸). Ideal thermal and mechanical parameters of 4H-SiC, diamond, sapphire and the PCB are considered for the simulation. In the case of 4H-SiC, the reduction of thermal conductivity at higher temperatures is incorporated, whereas device cooling through convection and through surface emittance is not. These assumptions, which are verified in vacuum at low device temperatures, result in a general overestimation of the device temperature, which thus represents an upper limit for the experimental conditions (Harris, 1995 ▸).

As X-ray beam we assumed those of the OPTICS beamline at SLS, which delivers a 100 µm FWHM Gaussian beam with total power density of 180 kW cm^−2^ corresponding to a power of 18 W and a photon energy distribution between 4 keV and 40 keV with a maximum at 15 keV. The absorption in 4H-SiC and diamond as a function of depth from the surface is calculated as
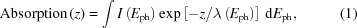
where *I*(*E*_ph_) and λ(*E*_ph_) are the beam intensity and attenuation length as a function of photon energy, *E*_ph_, respectively (Henke *et al.*, 1993 ▸).

As can be seen in Fig. 1(*b*) ▸, 4H-SiC shows a ‘hot-spot’ of 700°C at the center of the beam. The peak temperature in diamond is much lower (≥10×) due to its lower absorbed energy density (same absorption, thicker material) compared with 4H-SiC. It is worth noting that the maximum temperature obtained in 4H-SiC, 700°C, is still much lower than 1200°C, at which point the device would show intrinsic failure. Intrinsic failure is where thermally excited carriers dominate and create a leakage path between the different pads of the XBPM. It is also much lower than the melting point of the NiSi alloy (1300°C), which is used as a contact metal.

Furthermore, we find a maximum volumetric strain of ∼0.6% and less than 200 nm membrane deflection; neither of two results is critical in terms of mechanical device reliability. Finally, the temperature at the contacts (pads and PCB) is found to be below 100°C, so that 4H-SiC XBPMs should be stable and reliable even at such high operating temperatures. Nevertheless, further work is necessary to empirically prove the robustness of 4H-SiC XBPMs under such high-brilliance beams.

**Figure 2:**
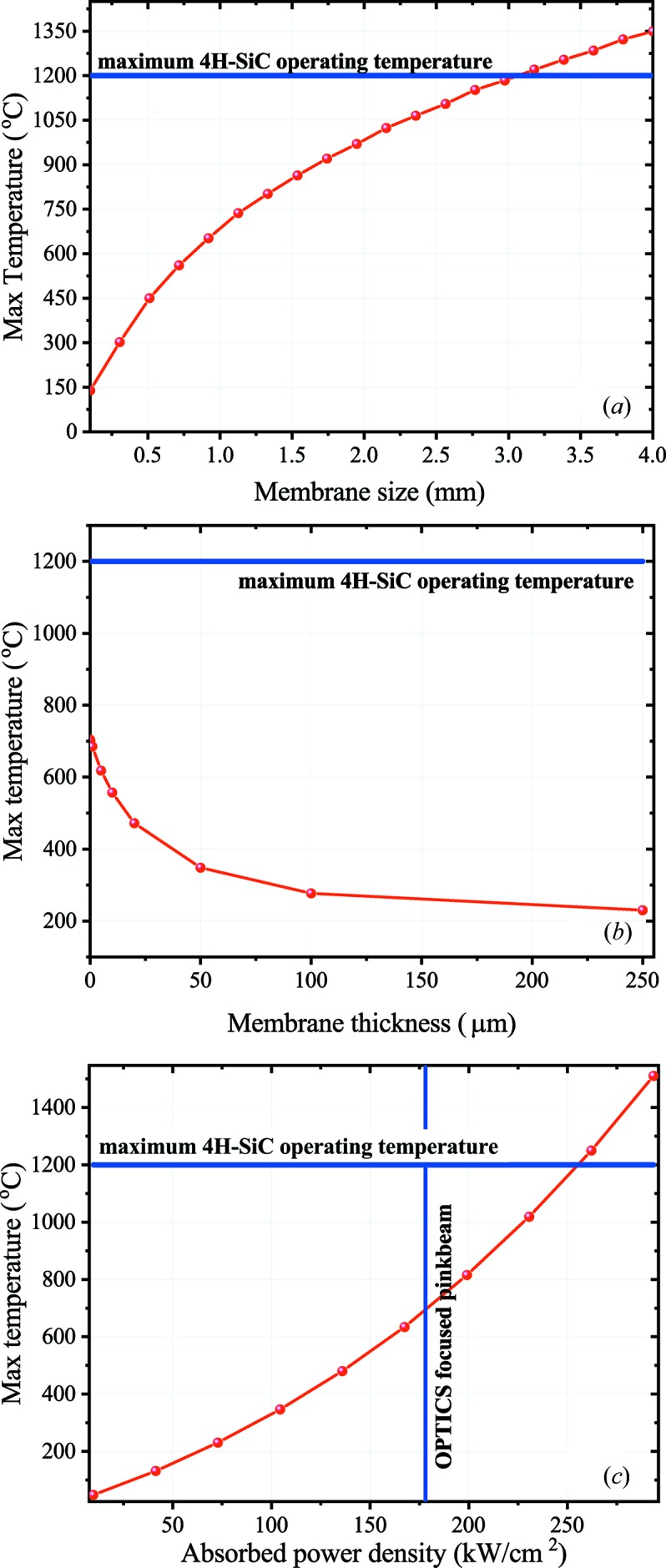
Maximum temperature as a function of (*a*) membrane size, (*b*) thickness of the membrane and (*c*) beam power density. When not swept, the simulations assumed 1 mm × 1 mm membrane size, 1 µm membrane thickness and 180 kW cm^−2^ beam power density. For such a device and beam characteristics, the absorbed power is 2.1% of the total beam power density.

In the system considered, the removal of the heat generated by the X-ray beam is achieved initially through the thin membrane and then through the substrate in contact with the cooled sapphire plate. For this reason, the device temperature increases for increasing membrane size [see Fig. 2(*a*) ▸] and decreases for increasing membrane thickness [Fig. 2(*b*) ▸]. Fig. 2(*c*) ▸ shows that a 4H-SiC XBPM on a 1.075 µm-thick and 1 mm × 1 mm large membrane can withstand up to 1.3 times the power density delivered by the OPTICS beam in focused pink beam configuration before reaching intrinsic failure of the device.

**Figure 3:**
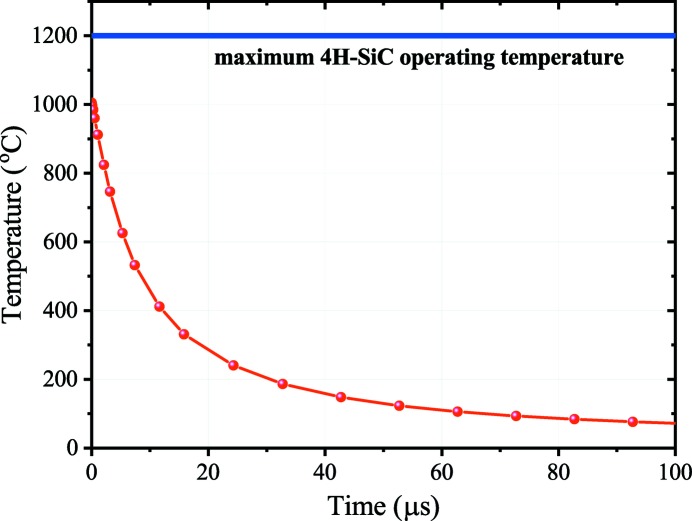
Temperature profile over time considering a 1.4 mJ, 12.7 keV, 10 fs-long beam with 50 µm FWHM, consistent with the ARAMIS SwissFEL beamline.

Finally, for the case of fast and high-power XFEL beams, such as the ARAMIS SwissFEL which delivers 1.4 mJ at 12.5 keV, 10 fs-long 50 µm FWHM ‘shots’ at 100 Hz, the temporal profile of the maximum temperature is also investigated. Simulations are made in vacuum assuming the absence of convective cooling. As shown in Fig. 3 ▸, the temperature on the surface of the XBPM in the center of the beam in such a case reaches a maximum of 1000°C during the femtosecond ‘shot’ but relaxes back to 100°C within 80 µs. The maximum temperature reached is, also in such a case, below the critical limit and, for a typical repetition rate of 100 Hz, the 4H-SiC XBPM has enough time to cool down. The maximum mechanical strain in this case is below 0.5%.

### 2.2. Electrical/optical simulations   

**Figure 4:**
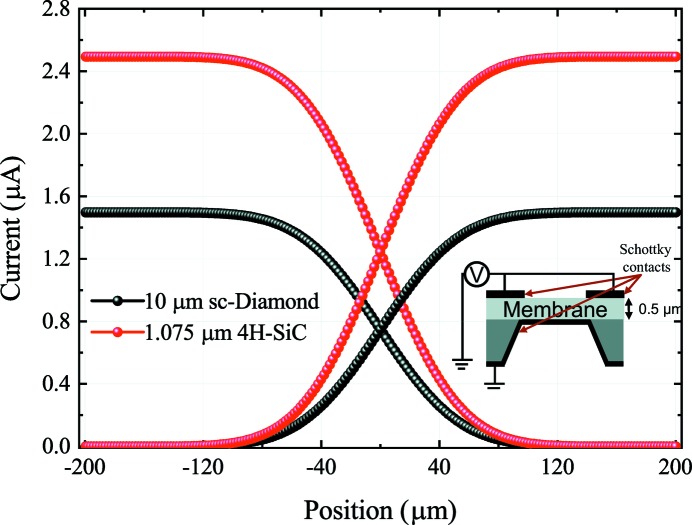
Collector current as a function of beam position for 20 V bias of sc-diamond and 4H-SiC. Position = 0 corresponds to the middle of the gap. The curves for both collectors are shown individually. The inset shows the cross section of the device (not drawn to scale; gap = 6 µm, membrane ≃ mm).

The electrical and optical performance of ideal 4H-SiC and sc-diamond Schottky barrier diode XBPMs are simulated using a TCAD software from Synopsys. A two-dimensional structure is sufficient to simulate the main aspects of XBPMs, namely the charge collection efficiency and the position sensitivity (which is related to the lateral resolution). An X-ray beam with 8 keV energy, a photon flux of 1 × 10^12^ photons s^−1^ and a FWHM of 100 µm is used for the simulation. The ideal sc-diamond XBPM consists of a 10 µm-thick nitrogen-doped (2 × 10^14^ cm^−3^) membrane with two Schottky contacts on the two sides (see inset of Fig. 4 ▸). A barrier height of 1.5 eV for a nickel contact is assumed at the collector and the emitter. Two collector pads, separated by a 6 µm gap, are placed on top and the emitter metallization covers the bottom. The device is simulated at 20 V bias.

Diamond is highly transparent to X-ray beams with a tenth of the absorption coefficient of 4H-SiC (Henke *et al.*, 1993 ▸). Therefore, to achieve the same transparency as a 10 µm diamond at 8 keV, a 1.075 µm-thick 4H-SiC is used. A nickel Schottky contact to 4H-SiC with a barrier height of 1.5 eV is assumed for both collectors and the emitter (Itoh *et al.*, 1995 ▸).

Self-consistent drift diffusion equations are solved for electrical transport along with the transfer matrix method (TMM) for photogeneration and propagation of the beam. Optical generation here assumes that the energy of absorbed photons is fully converted to excitation of valence electrons with an empirically determined ionization threshold of 7.8 eV in 4H-SiC and 13 eV in diamond (Bertuccio & Casiraghi, 2003 ▸). This results in an effective quantum yield, the number of electron–hole pairs generated per photon, of 1025 in 4H-SiC and 615 in sc-diamond for an incoming X-ray beam of energy 8 keV. These carriers either recombine *via* the Shockley–Read–Hall (SRH) process or are collected at the contacts. The SRH model includes doping-dependent lifetimes in 4H-SiC whereas, due to the lack of proper model parameters, the lifetime in sc-diamond is assumed to be constant (Table 1 ▸). In 4H-SiC, generation of carriers due to impact ionization is also taken into account by the Hatakeyama model (Hatakeyama, 2009 ▸). Material parameters used for the simulation are listed in Table 1 ▸. It is important to note that in diamond these values refer to the ideal high-purity sc-diamond whereas real devices, especially pc-diamond, are expected to perform significantly worse (Colbran, 2015 ▸).

Simulation of the beam scan is performed by moving the center of a Gaussian beam across the XBPM. In a horizontal beam scan, the photocurrent at the left collector goes from maximum when the beam is fully under this collector to minimum when the beam is fully under the right collector (Fig. 4 ▸). The maximum current depends on the beam intensity, and the effective quantum yield and collection efficiency of the device. A good XBPM generates a larger number of carriers per photon as well as effectively separating them towards the respective contacts before they recombine. It can be seen that the maximum current is about 63% higher in 4H-SiC than in sc-diamond. This difference is due to the difference in ionization energy, and thus effective quantum yield, between the two materials.

The position sensitivity, defined here by equation (2), is a measure of the change in current as a function of the beam motion,
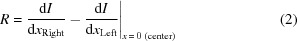
where *x* is the position of the beam, with zero in the center of the gap. The higher the position sensitivity, the smaller the resolvable beam movement. For a 100 µm beam under 20 V bias, the position sensitivity of a 4H-SiC XBPM (47 nA µm^−1^) is about 67% higher than that of a sc-diamond XBPM (28 nA µm^−1^).

**Figure 5:**
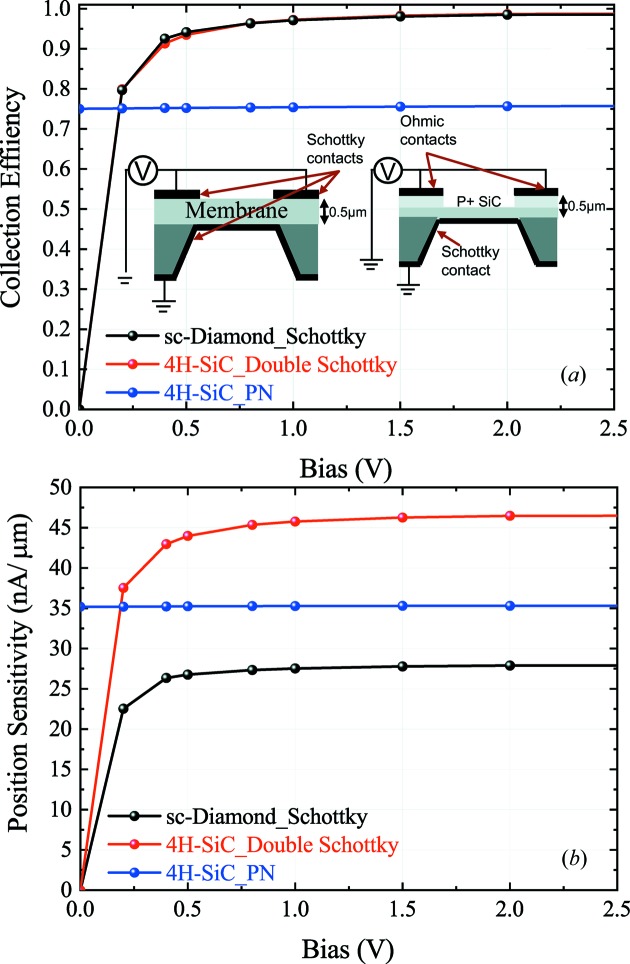
(*a*) Influence of the bias on collection efficiency for a beam position under the electrodes. The inset shows the device architecture (not drawn to scale). (*b*) Influence of the bias on position sensitivity.

To better understand the effect of bias on position sensitivity and collection efficiency of XBPMs, the actual device structure used in our experiments, a 4H-SiC p–n junction diode [inset of Fig. 5(*a*) ▸] is also added for comparison.

The collection efficiency of the three different structures is presented in Fig. 5(*a*) ▸. Both sc-diamond and 4H-SiC Schottky diodes need about 800 mV to gain ≥90% collection efficiency. This is because a bias is needed to drift carriers into the contacts. On the other hand, thanks to its built-in electric field, the p–n junction is able to collect its maximum efficiency at zero applied voltage. But this benefit comes at a cost of a degradation of the collection efficiency due to incomplete separation of the generated carriers in the heavily doped p+ layer.

When comparing the position sensitivity of the three structures, we see that the 4H-SiC Schottky diode has a 67% higher signal than the sc-diamond XBPM due to the lower ionization energy. However, the lower collection efficiency and slightly higher recombination in the p+ region results in only about 27% higher position sensitivity of the pn-junction compared with the sc-diamond.

## 3. Device fabrication   

4H-SiC XBPM devices are fabricated on wafers with epitaxial layers grown on 375 µm 1 × 10^18^ cm^−3^ n-type substrates. The epitaxial layers are either 2 µm- or 10 µm-thick 5 × 10^13^ cm^−3^ n-type with a 0.5 µm 1 × 10^18^ cm^−3^ p-type as a top layer. The four-quadrant monitors are fabricated with reactive ion etching (RIE) in SF_6_/argon plasma to remove the p-type layer with deposited metals as an etching mask. The etching mask is then used as an electrical contact to remove substrates *via* electrochemical etching. Different membrane thicknesses are achieved with further dry etching in SF_6_/Ar plasma.

Electrochemical etching (ECE) is an oxidation/oxide removal process obtained by dipping silicon carbide samples in an HF solution and electrically supplying holes for the oxidation through the back metal contact (Dahal *et al.*, 2017 ▸; Watanabe *et al.*, 2011 ▸; Gautier *et al.*, 2012 ▸, 2013 ▸). The process is capable of removing highly doped (≥1 × 10^18^ cm^−3^) p-type and n-type layers but is selective towards low-doped n-type layers (selectivity ≥ 1000:1 with respect to 5 × 10^13^ cm^−3^ doped layers). This allows the thick highly doped substrate to be selectively removed, realizing membranes with thicknesses and uniformities as determined by the epitaxial layers.

In this study, to evaluate devices with different thicknesses starting from only two epitaxies, some devices are further etched using a standard RIE SF_6_/Ar process. This allowed us to obtain devices with thicknesses down to 0.6 µm (0.5 µm p-type plus 0.1 µm n-type), as determined from the etching rate of the process. Consistent with the commercial pc-diamond device used for comparison (RIGI, DECTRIS), a 6 µm gap separated the four front electrodes [cross section shown in the inset of Fig. 5(*a*) ▸].

## 4. Measurement results   

Most of experimental tests are conducted at either the X06SA or at the OPTICS beamlines of the SLS at the PSI in Switzerland. Beam widths between 50 and 200 µm in both vertical and horizontal direction are obtained by two collimator slits located 5 mm before the XBPM. Transmission through the XBPMs is determined by comparing the current of a 300 µm Hamamatsu silicon diode monitor 35 cm downstream of the XPBM with and without the XBPM intercepting the beam. Motorized translation stages enabled precise movement of the XBPM in two orthogonal directions (*x*, *y*) transverse to the beam. The rear-side contact of the XBPM is biased and the current signals from the quadrant electrodes are measured by a four-channel electrometer (AH501D from CAENELS). Unless indicated otherwise, all measurements described below are performed with this configuration.

**Figure 6:**
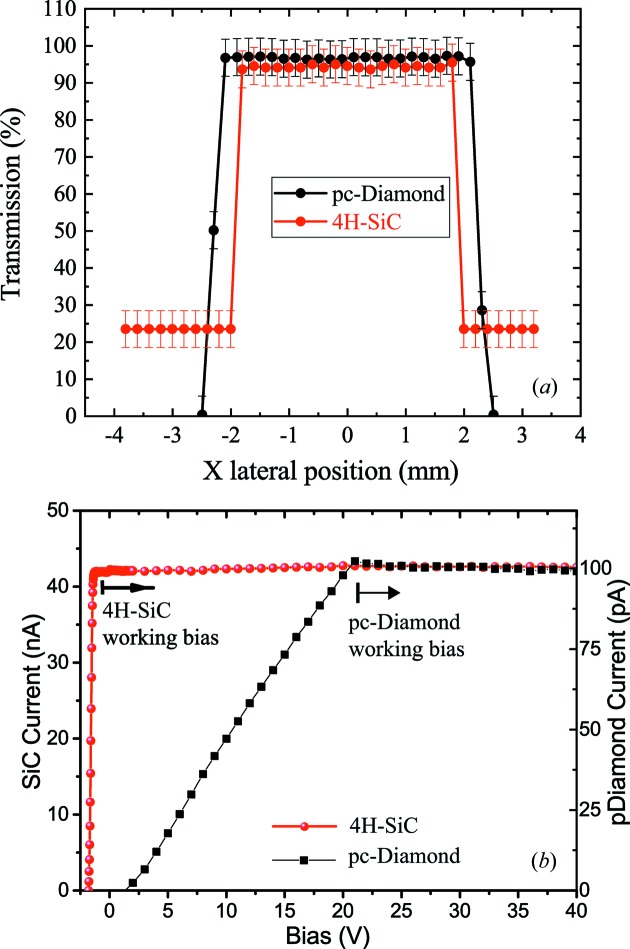
(*a*) Transmission as a function of the beam lateral position for the 1.24 µm 4H-SiC and 12 µm pc-diamond XBPMs using a 50 µm × 50 µm beam at 8 keV photon energy at the OPTICS beamline. The membrane shows 97% and 94% transmission (±5%) while the substrate is below 30 ± 5%. (*b*) Charge collection efficiency for the 10.5 µm 4H-SiC and pc-diamond showing 4H-SiC XBPMs collecting all carriers already at zero bias. Note that a 10.5 µm 4H-SiC is used here to allow external biases comparable with that used for pc-diamond.

Fig. 6(*a*) ▸ shows the beam transmission as a function of the beam lateral position for the 1.24 µm 4H-SiC and a 12 µm commercial pc-diamond XBPM device supplied by DECTRIS at 8 keV beam energy. Within the measurement error, systematic plus random, estimated to be ∼2 nA, equivalent to ∼5% transmission error, we observe a well defined membrane region in both devices. The transmission on the 4H-SiC membrane is ≥95%, compared with regions of the sample with the substrate where the transmission is below 30%. The 12 µm pc-diamond and 1.24 µm 4H-SiC XBPM have similar transmission within experimental error.

We subsequently analyse the electrical response of the 4H-SiC XBPM as compared with that of the pc-diamond. In the case of 4H-SiC, the built-in bias of the p–n junction in 4H-SiC achieves saturated charge collection at zero external bias, consistent with device simulations [Fig. 5(*a*) ▸]. On the other hand, saturated collection efficiency is achieved in pc-diamond only for biases greater than 20 V due to lifetime killing defects. Without these defects, sc-diamond requires only ∼2 V to achieve saturated collection [Fig. 5(*a*) ▸ and Desjardins *et al.* (2014 ▸)]. Given the above result, all measurements on 4H-SiC XBPMs are performed at zero bias and, consistently with vendor specifications, we will always use 30 V for pc-diamond.

**Figure 7:**
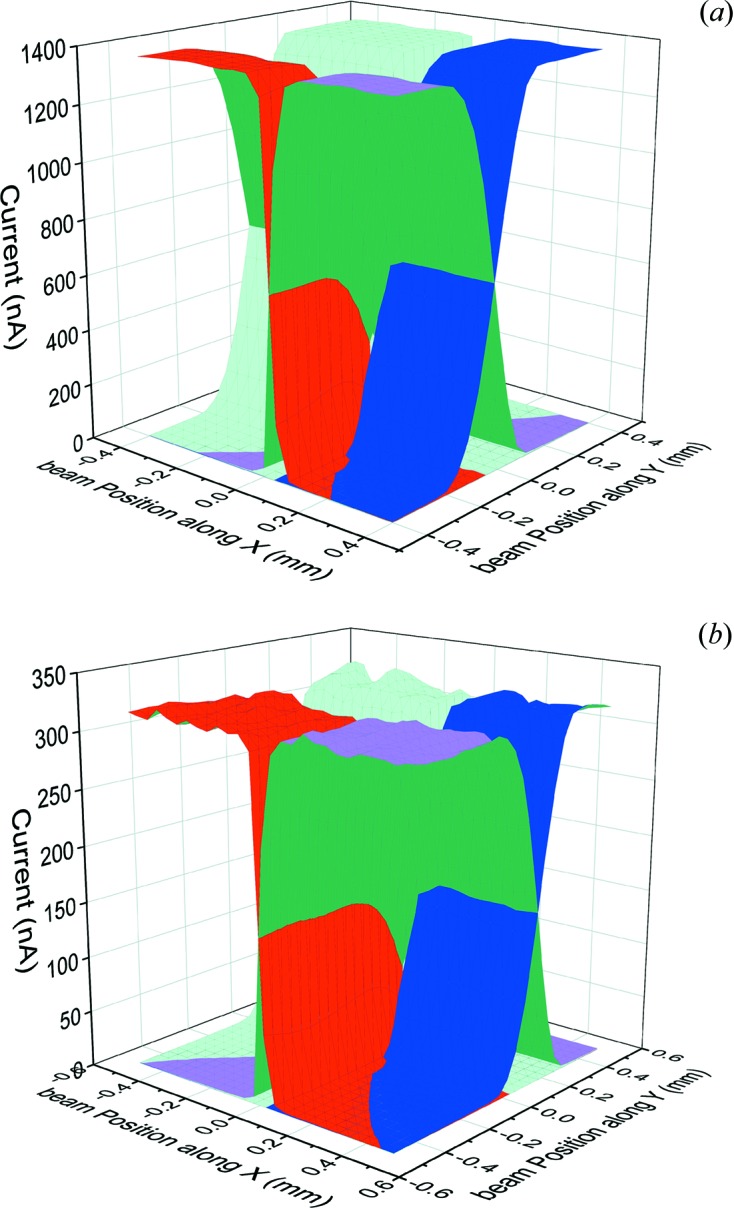
Current signal as measured from the different pads as a function of the beam position for the (*a*) 1.24 µm 4H-SiC XBPM and (*b*) 12 µm pc-diamond XBPM using a 12.4 keV photon energy and ∼200 µm 6 × 10^11^ photons s^−1^ flux beam at the X06SA beamline.

Fig. 7 ▸ shows the electrical response of the 1.24 µm 4H-SiC XBPM in *XY* raster scan as compared with that of pc-diamond. Both devices show good uniformity, but the 4H-SiC XBPM shows a superior, more than four times higher, signal-to-noise ratio. The noise level for both XBPMs was below the measurement limit of 100 pA.

The observed higher current signal for the 4H-SiC device is partially due to the difference in electron–hole pair creation energy of the two materials [7.8 eV and 13 eV for 4H-SiC and diamond, respectively (Bertuccio & Casiraghi, 2003 ▸)] and partially due to the difference in the charge collection efficiency of the two devices (91% and 31% for the 4H-SiC and pc-diamond, respectively, at 20 V, 12.4 keV, 6 × 10^11^ photons s^−1^).

**Figure 8:**
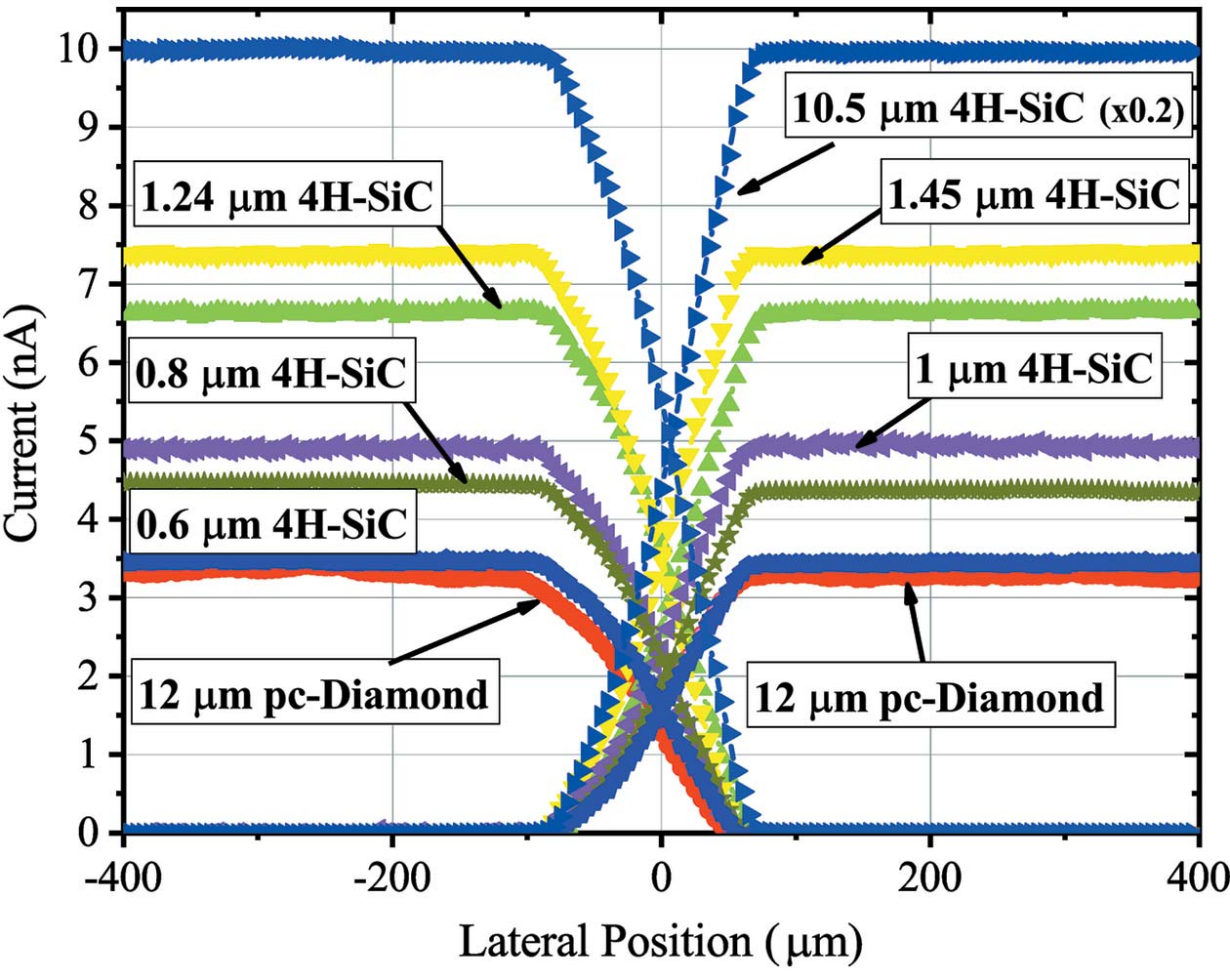
Current signal as measured from the different pads as a function of the beam position scanning along the horizontal direction using a 50 µm × 50 µm beam at 8 keV photon energy at the OPTICS beamline. Note that the 10.5 µm 4H-SiC XBPM signal is re-scaled to 20% of the actual value to give the reader a clear overview of all XBPMs.

Fig. 8 ▸ shows one-dimensional scans of a 100 µm X-ray beam along the *x*-direction for 4H-SiC devices with different thicknesses. Although all 4H-SiC devices show superior signal uniformity compared with pc-diamond, it shall be mentioned that, in the case of the very thin 4H-SiC XBPMs (≤1.1 µm), no fully functional four-quadrant device was obtained. The reason for such low yield can be the very thin low-doped n-layer, which prevents proper rectifying behavior.

**Figure 9:**
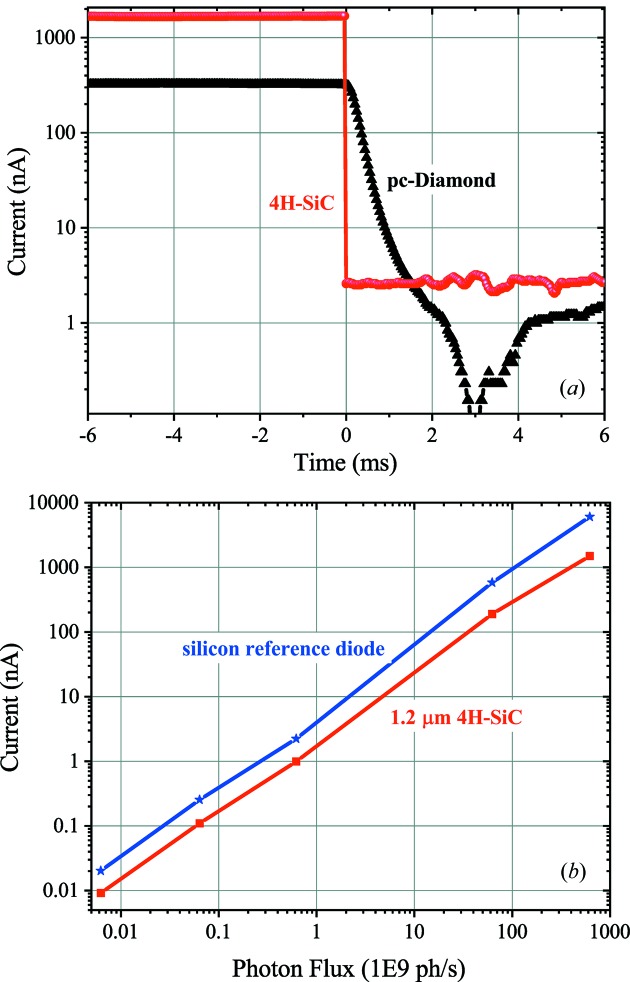
(*a*) Faster dynamics of the 4H-SiC XBPM (unbiased) compared with pc-diamond (biased at 30 V). (*b*) Linearity of 4H-SiC XBPMs in comparison with silicon diode. Both measurements are performed using a 12.4 keV photon energy and 6 × 10^11^ photons s^−1^ flux beam at the X06SA/PX1 beamline.

Finally, the response of the 1.24 µm 4H-SiC XBPM to variations in the photon flux is analyzed. The dynamic response of the XBPM is analysed by opening and closing the mechanical shutter of the X-ray beam whereas the current response for different photon fluxes is studied by using different filters along the optical path. The 4H-SiC XBPM shows much faster dynamics (in the microsecond range, currently limited by the measurement setup) compared with pc-diamond (millisecond range) [see Fig. 9(*a*) ▸]. In addition, it shows a linear response to photon flux for more than four orders of magnitude, similar to a reference silicon diode [see Fig. 9(*b*) ▸].

## 5. Conclusion   

In this paper, an extended simulation and experiments-based comparison between 4H-SiC and diamond XBPMs is presented.

Device simulations showed that, thanks to the lower electron–hole generation threshold energy, 4H-SiC has a potentially superior position sensitivity compared with even sc-diamond XBPMs under equivalent transmission. They also showed that, although 4H-SiC pin diodes can be operated at zero bias, they suffer from inferior charge collection efficiency due to higher recombination rates in the p+ region and inferior position sensitivity due to the reduced thickness in the gap between the front collectors.

Thermal simulations showed that even when the two materials absorb the same energy, with 4H-SiC at one-tenth of the thickness of sc-diamond, the higher absorbed energy density in 4H-SiC results in a larger increase in temperature. However, this high temperature is found to be below the critical operating temperature of 4H-SiC (1200°C) for the two experimental conditions: a 180 kW cm^−2^ (2.1% absorbed power) focused synchrotron pink beam and a 1.4 mJ, 10 fs, 12.4 keV monochromated XFEL beam, thus allowing applications of 4H-SiC even in the case of very high brilliance beams.

Preliminary experimental results based on the first fabricated 4H-SiC XBPMs compared with a commercial 12 µm pc-diamond device (RIGI, DECTRIS) showed uniform transparency across the device area. This excellent signal homogeneity shows the fabrication process to be suitable for producing highly uniform 4H-SiC membranes. Homogeneous and highly transparent membranes are prerequisites for precise beam-position monitoring and enable fast online position stabilization procedures. In addition, superior signal-to-noise ratio, linearity over four orders of magnitude of beam flux and faster dynamics (≤50 µs), at the limit of the current measurement setup, is achieved. The added possibility of operating the 4H-SiC XBPM without external biases potentially simplifies the signal processing circuit.

Given the obtained results and the maturity of this wide-band-gap semiconductor, we expect silicon carbide to substitute pc- and sc-diamond XBPMs in most beam-monitoring applications. Work is currently under way to further characterize 4H-SiC devices in terms of charge collection efficiency, radiation hardness and dynamics down to the nanosecond regime.

## Figures and Tables

**Table 1:** Device parameters used for simulation Optical parameters for both devices are from Henke *et al.* (1993 ▸) and electrical parameters for 4H-SiC from TCAD Sentaurus (2017 ▸). Material parameters for sc-diamond not listed below are taken from the Ioeffe database (http://www.ioffe.ru/SVA/NSM/Semicond/SiC/index.html).

Parameter	sc-Diamond electrons	sc-Diamond holes	4H-SiC electrons	4H-SiC holes
Lifetime (µs)	2 (Colbran, 2015 ▸)	2 (Colbran, 2015 ▸)	2.5	0.5
Saturation velocity (cm s^−1^)	1.9 × 10^7^ (Pomorski *et al.*, 2013 ▸)	1.52 × 10^7^ (Pomorski *et al.*, 2013 ▸)	1.9 × 10^7^ (Khan & Cooper, 2000 ▸)	1.9 × 10^7^
Mobility (undoped) (cm^2^ V^−1^ s^−1^)	2000 (Colbran, 2015 ▸)	2300 (Colbran, 2015 ▸)	1110	113.5 (Hatakeyama *et al.*, 2003 ▸)
